# Functional Magnetic Resonance Imaging Reveals Different Neural Substrates for the Effects of Orexin-1 and Orexin-2 Receptor Antagonists

**DOI:** 10.1371/journal.pone.0016406

**Published:** 2011-01-28

**Authors:** Alessandro Gozzi, Giuliano Turrini, Laura Piccoli, Mario Massagrande, David Amantini, Marinella Antolini, Prisca Martinelli, Nicola Cesari, Dino Montanari, Michela Tessari, Mauro Corsi, Angelo Bifone

**Affiliations:** 1 Center for Nanotechnology Innovation, Istituto Italiano di Tecnologia, Pisa, IIT@NEST, Italy; 2 GlaxoSmithKline Medicines Research Centre, Verona, Italy; Sapienza University of Rome, Italy

## Abstract

Orexins are neuro-modulatory peptides involved in the control of diverse physiological functions through interaction with two receptors, orexin-1 (OX1R) and orexin-2 (OX2R). Recent evidence in pre-clinical models points toward a putative dichotomic role of the two receptors, with OX2R predominantly involved in the regulation of the sleep/wake cycle and arousal, and the OX1R being more specifically involved in reward processing and motivated behaviour. However, the specific neural substrates underlying these distinct processes in the rat brain remain to be elucidated. Here we used functional magnetic resonance imaging (fMRI) in the rat to map the modulatory effect of selective OXR blockade on the functional response produced by D-amphetamine, a psychostimulant and arousing drug that stimulates orexigenic activity. OXR blockade was produced by GSK1059865 and JNJ1037049, two novel OX1R and OX2R antagonists with unprecedented selectivity at the counter receptor type. Both drugs inhibited the functional response to D-amphetamine albeit with distinct neuroanatomical patterns: GSK1059865 focally modulated functional responses in striatal terminals, whereas JNJ1037049 induced a widespread pattern of attenuation characterised by a prominent cortical involvement. At the same doses tested in the fMRI study, JNJ1037049 exhibited robust hypnotic properties, while GSK1059865 failed to display significant sleep-promoting effects, but significantly reduced drug-seeking behaviour in cocaine-induced conditioned place preference. Collectively, these findings highlight an essential contribution of the OX2R in modulating cortical activity and arousal, an effect that is consistent with the robust hypnotic effect exhibited by JNJ1037049. The subcortical and striatal pattern observed with GSK1059865 represent a possible neurofunctional correlate for the modulatory role of OX1R in controlling reward-processing and goal-oriented behaviours in the rat.

## Introduction

Orexins (hypocretins) are neuropeptides synthesized in the central nervous system by hypothalamic neurons [1]. Orexin-containing neurons interact with major modulatory neurotransmission systems and have been implicated in a wide range of physiological functions including feeding, arousal and sleep, neuroendocrine function, autonomic control and reward-processing [2]–[6]. Two orexin receptors (OX1R and OX2R) have been identified with distinct and largely complementary patterns of expression in the brain [7]. Recent pharmacological data and phenotypic characterisation of mice with genetic alterations of the orexin system point towards a possible functional specialization for the two receptor subtypes. Specifically, genetic and behavioural research has highlighted a role for the OX2R in the regulation of sleep/wake cycle and energy homeostasis [2], [3], [8], [9], while recent neuro-anatomical and pharmacological results suggest a putative contribution of the OX1R in modulating motivated behaviour and reward function [2], [10], [11].

A number of pharmacological tools have been developed to help investigate OXR function *in vivo*, and preliminary clinical assessment of the therapeutic potential of dual OX1R-OX2R antagonists for the treatment of insomnia have produced promising results [12], [13]. However, the limited selectivity of the OXR antagonists available has prevented a full elucidation of the relative neuro-functional contribution of each receptor sub-type on specific physiological functions of potential therapeutic relevance. Moreover, little is known of the circuital substrates modulated by the two receptors *in vivo*, and the relationship of these with specific behavioural outcomes.

In order to begin to address these questions, we used pharmacological magnetic resonance imaging (phMRI) to spatially-resolve the circuitry modulated by selective OXR antagonism in the rat brain. OX2R blockade was produced by using JNJ10397049 [9], [14], a recently described potent and selective OX2R antagonist, while OX1R blockade was induced with the novel OX1R antagonist GSK1059865 (5-bromo-N-[(2S,5S)-1-(3-fluoro-2-methoxybenzoyl)-5-methylpiperidin-2-yl]methyl-pyridin-2-amine) [15]. We show that the two compounds exhibit unprecedented selectivity (≥100-fold) over the counter receptor sub-type and display favourable pharmacokinetic properties which make them ideally-suited for *in vivo* rodent studies. The imaging studies were performed using the psychostimulant d-amphetamine to stimulate orexigenic activity [16], [17] and thus amplify the modulatory effect of OXR blockade independently of tonic levels of orexigenic activity. Finally, in an attempt to identify putative behavioural correlates of the imaging findings, the two compounds were tested in behavioural measures of sleep and reward-processing at the same doses used in the imaging experiments.

## Results

### In vitro potency and selectivity

Both GSK1059865 and JNJ10397049 antagonised, in a concentration dependent manner, Orexin-A-induced [^3^H] Inositol Phosphates (IP) accumulation (N = 3) in RBL cells stably expressing either rat OX1R or OX2R. GSK1059865 and JNJ10397049 displayed a non-surmountable antagonism of Orexin-A with depression of the agonist maximal response at OX1R. The pK_B_ values were 8.8 (CI_95%_ 8.7–9.1) and 5.9 (CI_95%_ 5.8–6.1) for GSK1059865 and JNJ10397049, respectively. On the contrary, the antagonism of the two compounds at OX2R was surmountable with a right shift of the agonist concentration response curve without depression of the maximal effect. The pK_B_ values were 6.9 (CI_95%_ 6.8–7.0) and 8.5 (CI_95%_ 8.3–8.6) for GSK1059865 and JNJ10397049, respectively. The potency values obtained for JNJ10397049 are consistent with previously published pK_i_ data [5.7 and 8.2, respectively, 14]. The same authors reported no significant affinity of JNJ10397049 in a panel of more than 50 other neurotransmitters and neuropeptide receptors (<50% inhibition at 1 µM, [14]). Likewise, GSK1059865 did not highlight significant affinity (<50% inhibition at 1 µM) over a panel of 113 receptors with the exception of κ-opioid (KOP) receptor for which GSK1059865 has a pKi of 6.5, corresponding to a selectivity of approximately 300 fold versus OX1R.

### Pharmacokinetic parameters and dose selection

**Figure 1:**
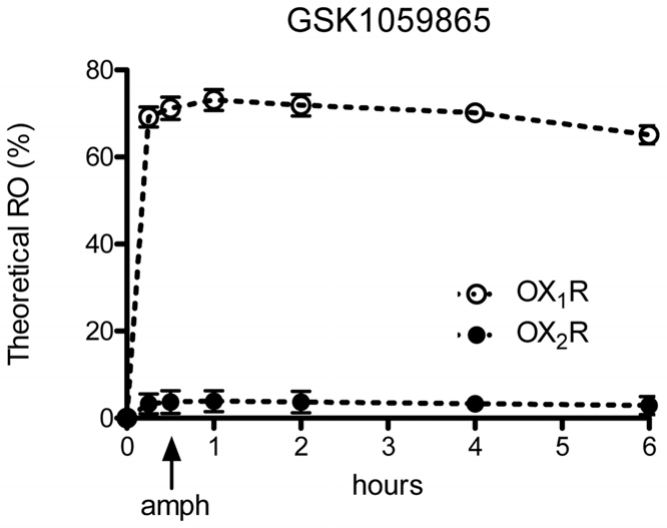
Brain receptor occupancy of GSK1059865. Theoretical brain receptor occupancy for GSK1059865 after intraperitoneal administration of 30 mg/kg to male SD rats. Data points represent mean +/− standard deviation.

In order to identify a suitable dose of GSK1059865 for the imaging studies, we measured pharmacokinetic parameters of the compound upon intraperitoneal administration, and used these to estimate the corresponding theoretical brain receptor occupancies. Pharmacokinetic analysis showed that the compound is brain penetrant (Brain:Blood ratio was ∼0.3) and that intraperitoneal administration ensures ample and sustained systemic exposure. Based on the pharmacokinetic data and orexin receptor potencies, we estimated that a dose of 30 mg/kg of GSK1059865 corresponds to >70% OX1R occupancy while retaining full selectivity (<5% OX2R) over the counter receptor sub-type (Figure 1). These values make this dose suited to probe *in vivo* the role of OX1R blockade with negligible risk of spurious OX2R contributions.

Pharmacokinetic parameters and *ex-vivo* OX2R occupancy in the rat brain of JNJ10397049 have been recently described [9]. Based on these data, for the imaging experiment we selected a dose of 50 mg/kg, corresponding to an OX2R occupancy higher than >70%. In the light of the high selectivity of the drug for OX2R over OX1R (400-fold) negligible receptor occupancy at the counter receptor sub-type is expected at this dose.

### OX1R and OX2R antagonism differentially attenuate the functional response to d-amphetamine

In order to spatially-resolve the neuronal substrates affected by selective OX1R or OX2R antagonism in the rat brain, we mapped the modulatory effect of GSK1059865 and JNJ10397049 in animals challenged with d-amphetamine, a drug that stimulates orexigenic activity, using phMRI.

**Figure 2:**
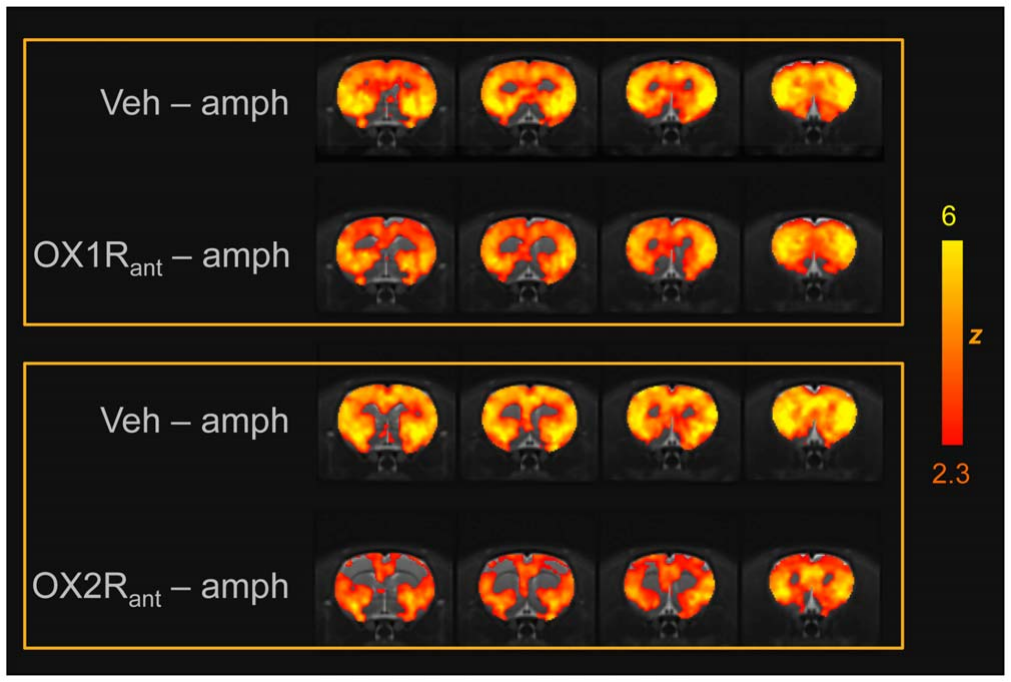
fMRI activation produced by D-amphetamine as a function of pharmacological pre-treatment. Anatomical distribution of the activation produced by D-amphetamine challenge across the different treatment groups. Yellow/orange indicates increased rCBV versus vehicle-vehicle baseline (Group 5). OX1R_ant_: GSK1059865 (30 mg/kg i.p.); Ox2R_ant:_: JNJ10397049 (50 mg/kg i.p.).

Consistent with previous studies [18] d-amphetamine produced robust, widespread and sustained rCBV increases in cortical and subcortical regions (Figures 2–4). The effect is unlikely to be dominated by peripheral blood pressure changes, as these were not temporally correlated with the rCBV time-course (Figure S2), and well within the blood flow autoregulatory range within which vasopressive responses are homeostatically compensated without producing significant rCBV alterations [19].

**Figure 3 pone-0016406-g003:**
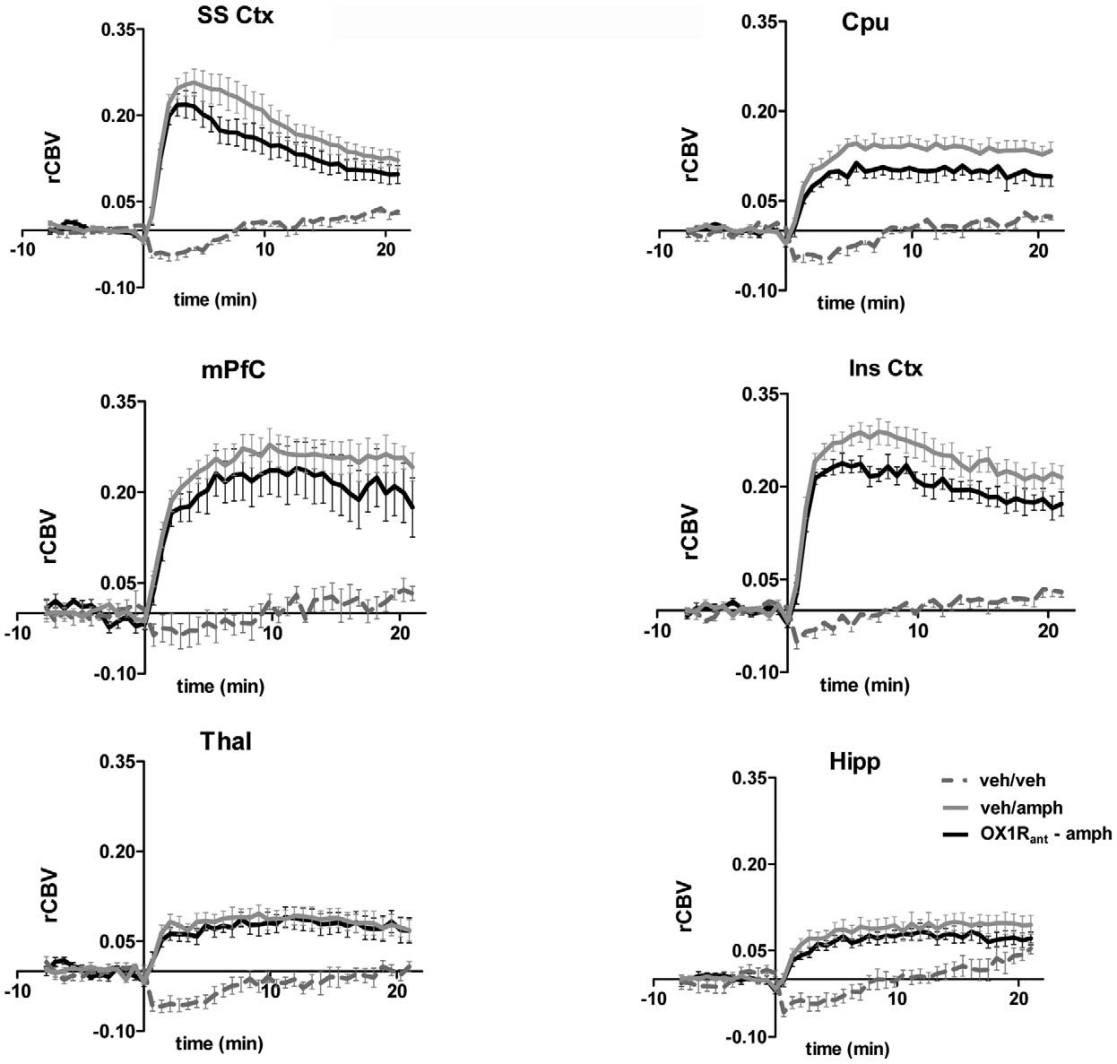
fMRI timecourse in representative brain regions: effect of OX1R antagonism. Temporal-profile of amphetamine-induced rCBV changes in representative VOIs. Data are plotted as mean±SEM within each group. OX1R_ant_: GSK1059865 (30 mg/kg i.p.) [SS Ctx: somatosensory cortex; Cpu: caudate putamen; mPFC: medial prefrontal cortex; Ins Ctx; insular cortex; Thal; thalamus; Hipp: hippocampus].

**Figure 4:**
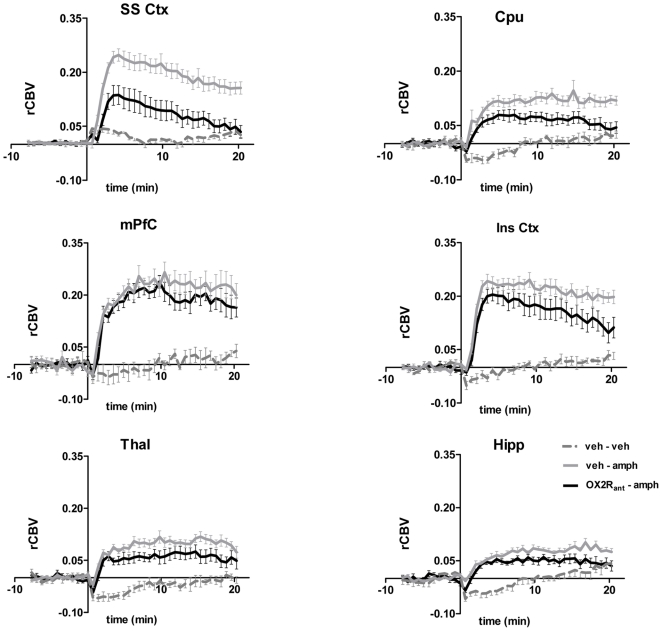
fMRI timecourse in representative brain regions: effect of OX2R antagonism. Temporal-profile of amphetamine-induced rCBV changes in representative VOIs. Data are plotted as mean±SEM within each group. OX2R_ant:_: JNJ10397049 (50 mg/kg i.p.) [SS Ctx: somatosensory cortex; Cpu: caudate putamen; mPFC: medial prefrontal cortex; Ins Ctx; insular cortex; Thal; thalamus; Hipp: hippocampus].

**Figure 5:**
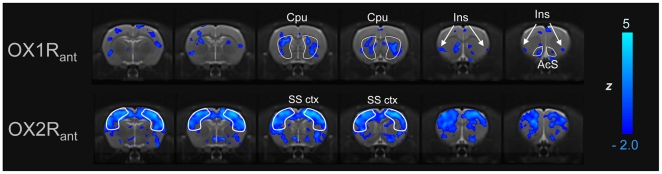
Attenuation of fMRI response by OX1R or OX2R antagonism. Regions of reduced amphetamine-induced rCBV response following pre-treatment with GSK1059865 (OX1R_ant_; top) or JNJ10397049 (OX2R_ant_, bottom). Blue indicates *reduced* rCBV response versus control (vehicle-amphetamine). Cpu: caudate putamen; SS ctx: somatosensory cortex; Ins: insular cortex, AcS: shell of the nucleus accumbens.

Pretreatment with the selective OX1R antagonist GSK1059865 (30 mg/kg i.p) produced a significant attenuation of d-amphetamine's functional response in sub-cortical areas and focal cortical regions (Z>1.96, Figures 2, 3 and 5). The effect was bilateral and prominent in the dorso-lateral striatum but extended also to the fronto-ventral areas of this structure up to the frontal portions of the shell of the nucleus accumbens (Z>1.96, Figure 5). Among cortical regions, the insular, cingulate and primary auditory cortices were the only areas that exhibited significant attenuation (Figures 3 and 5). Occasional small spots of inhibition were also observed in the rostral portions of the somatosensory cortex. By contrast, pretreatment with the selective OX2R antagonist JNJ10397049 (50 mg/kg i.p) produced a substantially more widespread pattern of attenuation that included most cortical regions (Figures 2, 4 and 5), with substantial contributions from somatosensory, motor, cingulate and orbitofrontal cortices. Weaker but significant attenuating effects were observed in sub-cortical nuclei of the thalamus, hypothalamus, and striatum and hippocampus (Figure 5).

No region showed increased response to d-amphetamine in animals pre-treated with either GSK1059865 or JNJ10397049. The lack of potentiating effects cannot be attributed to the use of an amphetamine dose producing “ceiling” functional effects, as region-specific potentiation of response by different pharmacological mechanisms has been previously described using the same protocol and dose of D-amphetamine [18], [20]. Neither GSK1059865 nor JNJ10397049 produced substantial rCBV alterations *per se* in any of the regions examined (Figures S3 and S4), ruling out potential confounding effects of baseline rCBV changes induced by either drug.

### OX2R but not OX1R antagonism produces robust sleep-promoting effects

**Figure 6:**
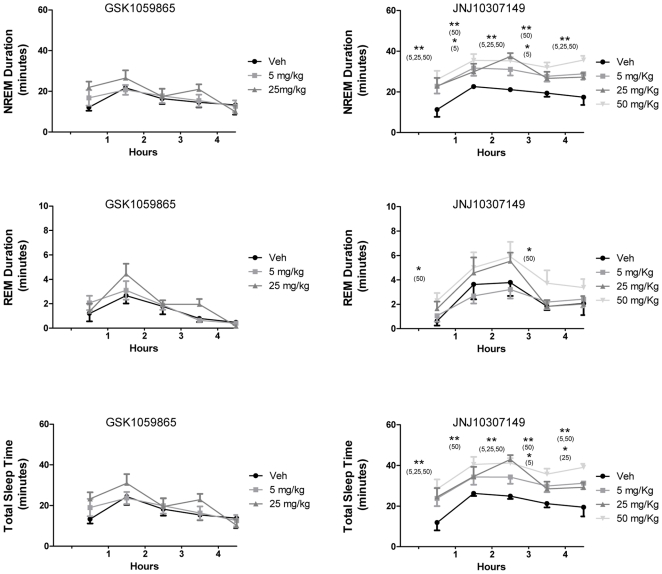
Sleep-promoting effects of selective OX1R or OX2R antagonism. Sleep-promoting effects (NREM, REM and total sleep time) of selective OX1R (Left; GSK1059865, 5 and 25 mg/kg i.p.) or OX2R antagonism (Right; JNJ-10397049; 5, 25, and 50 mg/kg) *versus* vehicle. Data are expressed in minutes and are represented as means ± S.E.M. Values were rebinned in 1-hour intervals to cover the whole 5-hour time-window recorded. (*, P<0.05 and **, P<0.01, treatment versus vehicle, on one-way ANOVA followed by Newmann-Keuls test). Doses that produced statistically significant effects are indicated in parenthesis above the corresponding time-point.

In an attempt to identify a behavioural correlate to the divergent imaging profile of the two compounds, we measured the sleep-promoting effect of GSK1059865 and JNJ10397049 when administered at 6 hrs into the dark phase, at time point at which the Orexin-A peptide is highly expressed [21]. JNJ10397049 showed profound sleep-promoting effects at all doses tested (5, 25 and 50 mg/kg i.p.). More specifically, JNJ10397049 substantially increased REM, NREM and total sleep-time across the whole 5 hour time-window of recording at all doses tested (Figure 6). Interestingly, the sleep-promoting effects of JNJ10397049 were not observed with GSK1059865. When tested at doses similar to those used in phMRI experiment assays (25 mg/kg i.p.), the compound did not significantly increase REM and total sleep-time over the 5 hours duration of the experiment, either as individual 1 hour bins or cumulative five-hour integration (p>0.16, all parameters, Figures 6), but only produced a small (<3 min) significant increase in a cumulative measure of total REM sleep time (p<0.02). At a lower dose (5 mg/kg i.p.) the compound did not significantly alter any of the sleep parameters recorded (Figure 6).

### OX1R antagonism dose-dependently reduces expression of cocaine-induced conditioned place preference (CPP)

**Figure 7:**
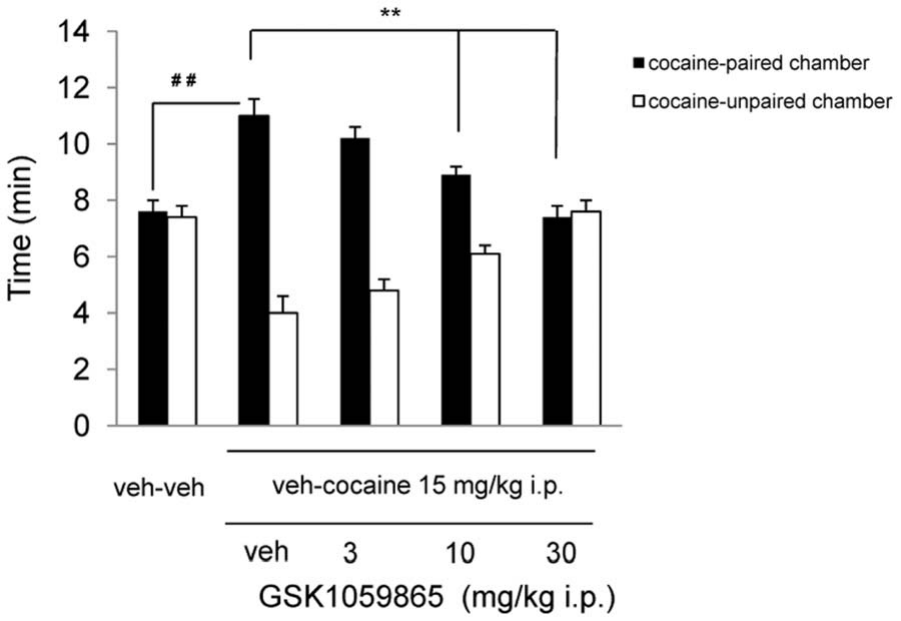
Effect of the OX1R antagonist on cocaine induced CPP. Effect of vehicle (veh) or GSK1059865A on the expression of cocaine-induced CPP response. Acute administration of 10 and 30 mg/kg i.p. of GSK1059865 significantly decreased the expression of the CPP response to cocaine compared to vehicle at 10 mg/kg and 30 mg/kg i.p. (** p<0.01 vs. Vehicle; ANOVA and Student-Newman-Keuls test). Times spent in the cocaine-paired and cocaine-unpaired chambers on test day are shown as means ±S.E.M. (## p<0.01 versus Vehicle-Cocaine + Vehicle on test day, Student's u npaired t test).

In order to investigate a possible behavioural correlate of the striatal phMRI effects observed with GSK1059865, we also tested the compound in a cocaine-induced conditioned place-preference (CPP) paradigm. This behavioural test is widely employed to measure drug-seeking (i.e. a goal-oriented behaviour) in laboratory animals [22] and crucially involves the recruitment of striatal dopaminergic areas as effectors of the reward-related processing underling the preference for the drug-paired chamber [23]. In keeping with the results obtained by other investigators using less selective OX1R antagonists [i.e. SB334867, 6,10], GSK1059865 dose-dependently inhibited cocaine-induced place preference, an effect that reached statistical significance at both 10 and 30 mg/kg i.p. (Figure 7). JNJ10397049's robust hypnotic and sedative profile at doses as low as 3 mg/kg prevented us from testing the drug in the same paradigm.

## Discussion

We have used fMRI to map the functional substrates of OX1R or OX2R blockade in the rat brain using GSK1059865 and JNJ1037049, two novel and highly-selective OXR antagonists. The imaging findings highlighted a different neuroanatomical pattern of functional inhibition for the two compounds: GSK1059865 produced focal striatal effects, whereas JNJ1037049 induced a composite pattern of modulation characterised by a prominent cortical involvement. Moreover, at doses similar to those tested in the fMRI study, JNJ1037049 exhibited robust hypnotic properties, whereas GSK1059865 did not display significant sleep-promoting effects. Collectively, these findings highlight an essential contribution of the OX2R in modulating cortical brain function, an effect that correlates with the robust hypnotic effect exhibited by JNJ1037049. The striatal effect observed with GSK1059865 represent a putative neurofunctional correlate for the recognised modulatory role of OX1R antagonism in reward-processing and goal-oriented behaviour in the rat, an effect that was confirmed with GSK1059865 in a cocaine-induced CPP paradigm.

In order to image the effects of OXR antagonism in anaesthetised animals, we developed a phMRI protocol relying on the use of the psychostimulant D-amphetamine. The drug elicits a composite pattern of activation involving cortical structures, reflecting the arousing and stimulating properties of the compound, as well as subcortical regions pivotally involved in processing of reward and control of motivated behaviour [24]. Moreover, amphetamines robustly activate orexin neurons [16], [17], and can be used to probe the modulatory action of OXR antagonism independently of tonic levels of orexigenic activity (i.e. in anaesthetised animals). We used rCBV as surrogate haemodynamic readout for underlying changes in brain activity [25], [26]. This measure has gained acceptance as the measure of choice in small animal fMRI studies where sensitivity is a significant technical challenge [27], [28].

The robust sleep-promoting effect of JNJ1037049 is consistent with recent pharmacological data [9] and with phenotypic characterisation of mice with genetic alterations of the OX2R system [2], [4]. Our sleep-measurements substantiate the central role of the OX2R in regulating arousal and the sleep\wake cycle. Within the framework of a divergent sleep-promoting effect of JNJ1037049 and GSK1059865, the imaging findings are of interest as they reveal a predominant contribution of the OX2R in controlling brain function in broad cortical and sub-cortical networks, an effect that might be functionally related to the hypnotic effect exhibited by JNJ1037049. Notably, positron emission tomography (PET) measurements of cerebral blood flow during physiological sleep in humans [reviewed in 29] have consistently shown coordinated inhibition of thalami, basal ganglia, hypothalamus and large neocortical areas, in a pattern that is qualitatively very consistent with the inhibition observed with JNJ1037049 in the present study. Given the rich and complex interactions between orexin and other neurotransmitter systems [reviewed in 2], the widespread functional inhibition produced by JNJ1037049 may integrate downstream contributions of heterogeneous nature that could involve multiple modulatory neurotransmitters including histamine, serotonin, noradrenaline, acetylcholine and dopamine. The availability of brain penetrant and selective tools like JNJ1037049 is expected to promote the investigation of the neurobiological and therapeutic significance of such interactions in normal and disease states.

The virtual absence of sleep-promoting effects of the selective OX1R antagonist GSK1059865 at the doses tested in the present study (i.e. with negligible OX2R occupancy) provides an independent confirmation of the pharmacological data reported by Dugovic et al., [9] showing that blockade of OX2R is sufficient to initiate and prolong sleep. Previous studies reported on the lack of significant change in the amount of sleep-wake states after administration of the OX1R antagonist SB-334867[30]. Taken together these studies point towards a role for the development of selective OX2R antagonists as functionally-specific sleep-promoting pharmacological tools.

Separate roles for the two OXR subtypes on sleep architecture that fit well with their differential distribution have been postulated; namely, a specific role in the regulation of REM sleep for OX1R in the locus coeruleus [30] and an implication of hypothalamic OX2R in the modulation of NREM sleep [4]. The observation of focal contribution of OX1R in modulating total REM sleep correlates with electroencephalographic phenotyping of orexin double-receptor (OX1R and OX2R) knockout which significantly differs from single OX2R -knockout by a greater incidence of direct transitions from wakefulness to REM sleep [4], [31]. Our data suggest that pharmacological OX1R may exert weak but significant influence on the duration of REM sleep even in presence of a functional OX2R.

An interesting finding of this study is the region-specific modulation of the striatum and focal cortical terminals observed with the OX1R antagonist. This pattern contrasts sharply with widespread and robust cortical deactivation observed with the OX2R antagonist JNJ1037049. Although the exact neuro-circuital cascade underlying the divergent imaging findings remains unknown, several hypotheses can be made in the light of previous anatomical and functional studies. Firstly, the focal deactivation of striatal terminals produced by GSK1059865 cam be related to the existing evidence of the OX1R in the control of reward processing and drug-seeking. Using a self-administration paradigm, Aston-Jones and colleagues [6], discovered that pre-treatment with the OX1R antagonist SB334867 blocks cocaine-seeking induced by discrete or contextual cues. The same drug significantly attenuated the expression of a morphine CPP [10]. However, SB334867 possesses micromolar or sub-micromolar affinity at other receptor types (i.e. adenosine_2A_R, pK_i_ = 7.2; melatonin_2_R pK_i_ = 6.9, serotonin_2C_ and norepinephrine transporter pK_i_ = 5.7; M. Corsi, unpublished results) which significantly narrows the selectivity window (<10-fold) over OX1R (pKB = 7.5) and poses questions as to whether the effects reported so far with SB334867 can be solely attributed to OX1R antagonism. Our cocaine CPP study with GSK1059865, a compound of unprecedented OX1R selectivity and structurally unrelated to SB334867, suggests that OX1R is sufficient to modulate reward-processing and drug-seeking behaviours. Interestingly, the CPP paradigm crucially involves the recruitment of striatal dopaminergic areas as effectors of the reward-related processing underling the preference for the drug-paired chamber [23]. Given the tight correlation between striatal response to D-amphetamine and local dopamine release [reviewed in 32], it is therefore tempting to speculate that the striatal inhibition observed with the OX1R antagonist may represent an indirect neurofunctional correlate of the ability of GSK1059865 to reduce expression cocaine-induced CPP. Such an effect would be consistent with the presence of major orexigenic projections to the ventral tegmental area [33] as well as the substantia nigra [34] and with the dramatic increase in orexin firing that has been observed in association to explorative behaviours [35], [36]. Such an hypothesis would support a theoretical construct where orexin neurons are strategically positioned at a transitional point where internally driven states (e.g desire for drugs), are translated into patterned motor response [37]. Within this framework, the presence of focal and bilateral deactivation in the insular cortex in the OX1R antagonist group is of great interest, given the central role of this structure as integrator of interoceptive states into conscious feelings and decision-making processes that precipitate relapse [38]. The involvement of OX1R in these processes has been recently supported by the results of a recent study [11] where pharmacological blockade of OX1R receptors in the insula, but not in the adjacent somatosensory cortex, inhibited nicotine intake in a self-administration paradigm. Collectively, and in agreement with the behavioural data, the imaging findings argue for a targeted modulatory role of the OX1R on neurobehavioural substrates that may be perturbed in states of addiction. These features make OX1R an attractive target for the development of novel pharmacological treatment for these disorders.

A few cautionary statements should be made about this study. Whether the circuits identified in the phMRI study underpin the homeostatic substrates modulated by OXRs, or rather are a consequence of the hyper-arousal state produced by the d-amphetamine is an aspect that could not be addressed due to the need to keep animals under anaesthesia to reduce stress and motion artefacts. Together with a possible interfering role of anaesthesia with tonic orexigenic activity, the use of d-amphetamine might have resulted in supra-physiological contributions that may limit the physiological relevance of the imaging findings to the homeostatic state. As selective orexin antagonists will become available for clinical investigations, a definite answer to this question will be provided by neuroimaging studies in conscious human subjects. It is however unlikely that the imaging findings reflect major unspecific pharmacological interactions between the OXR antagonists and the anaesthetic, as these would be expected to present themselves in the form of detectable rCBV alterations upon the injection of the pre-treatments *per se*. Moreover, halothane anaesthesia has been recently demonstrated to exert negligible influence on orexigenic activity [39].

In this study we did not measure the ex-vivo receptor occupancy of GSK1059865 and JNJ1037049 directly, but used estimates based on PK data and in vitro potencies. However, the high potency and selectivity of the two drugs argue against a spurious contribution of the counter-type receptors at the doses used for these studies. This notion is corroborated by the good correspondence between our behavioural results and those obtained by other authors under similar dosing regimens [9]. Moreover, additional imaging experiments performed in our lab with dual OXR antagonists characterised by different relative affinity at OX1R and OX2R confirmed the prevalence of cortical and striatal modulation patterns as a function of relative OX2R or OX1R occupancy, respectively (A. Gozzi, unpublished results). Another limitation is the use of a single-dose of each compound in the imaging study, a choice dictated by the complex and time consuming preparation and monitoring procedures used to ensure a tight control of animal physiology during the phMRI experiments. Clearly, an exploration of the imaging effects across a range of doses would be desirable and would in principle enable a better appraisal of the relative functional contribution of the two receptors. However, this would have lengthened the duration of the experiment considerably, at the expense of the homogeneity of the experimental groups of animals in terms of age, size and batch, with potentially detrimental effects. Finally, we point out that a trend for weak signal attenuation in cortical areas was also observed with GSK1059865 (Figure 3). Whether this effect is the result of inter-group statistical fluctuations, non-negligible levels of OX2R occupancy by GSK1059865, or functionally meaningful contributions of the OX1R is an aspect that deserves further consideration. However, the difference in the cortical effects of the two compounds in terms of both magnitude and spatial extension is large, and strongly support the conclusion of a preferential cortical control by the OX2R.

In summary, we mapped the neuronal substrate for the effects of selective OX1R or OX2R antagonism in the rat, and correlated the imaging changes with putative behavioural correlates. Our results reveal an essential contribution of the OX2R in modulating cortical activity and the sleep/wake cycle. Conversely, the subcortical pattern observed with GSK1059865 is suggestive of a striatal involvement in the control of reward-function and goal-oriented behaviours by the OX1R.

## Methods

### Ethics Statements

All the works involving animals were carried out in accordance with European directive 86/609/EEC governing animal welfare and protection, which is acknowledged by Italian Legislative Decree no. 116, 27 January 1992, and were reviewed and approved by the GlaxoSmithKline Committee on Animal Research & Ethics (CARE) under protocol #20094 according to the company Policy on the Care and Use of Laboratory Animals.

### Drugs

GSK1059865 and JNJ10397049 were synthesised by the GSK department of medicinal chemistry.

### In vitro potency and receptor specificity

The *in vitro* potencies of GSK1059865 and JNJ10397049 at rat orexin receptors were determined using a method previously described [40]. Briefly, RBL cells stably expressing either rat OX1R or OX2R receptor were seeded in 96-well plates (3×10^4^ or 1.5×10^4^ for OX1R and OX2R study, respectively) and supplemented with medium containing 1 µCi per well of [^3^H]-myo-inositol 16 hours prior to the beginning of the assay. Cells were then washed with the assay buffer (1X HBSS buffer, 20 mM HEPES pH 7.4, 0.1% BSA, 10 mM LiCl) and pre-incubated for 30 minutes at 37°C with different concentrations of JNJ10397049 (0.333–33.3 µM and 10 nM-0.333 µM at rat OX1R and rat OX2R, respectively) or GSK1059865 (0.1–33.3 nM and 0.1–3.33 µM at rat OX1R and rat OX2R, respectively). Orexin-A concentration-response curves (from 10 µM to 0.1 nM) were determined in the presence of LiCl 10 mM and 0.1% Bovine Serum Albumine over a 60-minute time-window. After cell lysis (0.1 M ice-cold formic acid), 20 µl of the supernatant were placed in 96 well Optiplate containing 1 mg/well Ysi RNA beads (Amersham) and shaken for 1 h at room temperature. After 2 h at 4°C, plates were read with a Packard TopCount scintillation counter. Data were analyzed with GraphPad prism (v.5.0) software. Antagonist potency values (pK_B_) were determined by using the operational model for apparent non surmountable compounds, or by applying Schild's analysis for compounds displaying surmountable profiles [41], [42]. pK_B_ values together with corresponding 95% of confidence interval were calculated from three independent experiments.

Receptor specificity for GSK1059865 was measured in a CEREP/Bioprint™ (CEREP, Redmond, WA) binding assay of 113 receptors, ion channels and transporters, where the drug was tested at a dose of 1 µM. The selectivity panel consisted of the following receptors (human isoforms): A_1_, A_2A_, A_3_, α_1_ (non-selective), α_2_ (non-selective), β_1_, β_2_, AT_1_, AT_2_, BZD (central), BZD (peripheral), B_1_, B_2_, CGRP, CB_1_, CB_2_, CCK_A_ (CCK_1_), CCK_B_ (CCK_2_), CRF, D_1_, D_2_, D_3_, D_4_, D_5_, ETA, ETB, GABA (non-selective), GAL_1_, GAL_2_, AMPA, Kainate, NMDA, Glycine (strychnine-insensitive), PDGF, CXCR2 (IL-8B), TNF-α, CCR_1_, CCR_2_, CCR_3_, Ghrelin (GHS), H1, H2, H3, ILTB4 (BLT1), LTD4 (CysLT1), MC4, MT_1_, M_1_, M_2_, M_3_, M_4_, M_5_, NK_1_, NK_2_, NK_3_, Y_1_, Y_2_, NT_1_ (NTS1), NmU2, N (neuronal) (α-BGTX-insensitive) (α4β2), δ2 (DOP), κ(KOP), μ (MOP) (agonist site), ORL1 (NOP), PACAP (PAC1), PPARγ, PAF, PCP, EP4, TXA2/PGH2, (TP) –platelets, IP (PGI2), P_2X_, P_2Y_, 5-HT_1A_, 5-HT_1B_, 5-HT_1D_, 5-HT2A, 5-HT_2B_ (agonist site), 5-HT_2C_, 5-HT_3_, 5-HT_4_, 5-HT_5A_, 5-HT_6_, 5-HT_7_, σ(non-selective), sst (non-selective), Glucocorticoid (GR), Estrogen, Progesterone (PR), Androgen (AR), TRH_1_ (M), UT1, VIP1 (VPAC1), V_1a_, V2,Ca^2+^ channel (L, DHP site), Ca^2+^ channel (L, diltiazem site) (benzothiazepines), Ca^2+^ channel (L, verapamil site) (phenylalkylamines), Ca^2+^ channel (N), K^+^ ATP channel, K^+^ V channel, SK^+^ Ca channel, Na channel (site 2), Cl channel, NE transporter, DA transporter, GABA transporter, Choline transporter (CHT1), 5-HT transporter. Selectivity data for JNJ10397049 have been described in previous reports [9,compound 9 in 14] where the drug was tested at 10 µM over the following panel of 50 neurotransmitter and neuropeptide receptors (Cerep ExpressProfile).: A_1_, A_2A_, A_3_, α1 (non-selective), α_2_ (non-selective), β_1_, β_2_, CB_1_, D_1_, D_2_, GABA (non-selective), BZD, Cl-channel (GABA-gated), H_1_, H_2_, MT_1_, M_1_, M_2_, M_3_, TP, 5-HT_1A_, 5-HT_1B_, 5-HT_2A_, 5-HT_2B_, 5-HT_3_, 5-HT_5A_, 5-HT_6_, 5-HT_7_, AT_1_, B_2_, CCR_1_,CXCR2, CCK_1_, ET_A_, GAL_2_, MC4, NK_2_, NK_3_, Y_1_, Y_2_, NT_1_ (NTS_1_), δ2 (DOP), κ(KOP), μ(MOP) (agonist site), NOP, sst, VPAC_1_, V_1a,_Ca^2+^ channel (L, DHP site), Ca^2+^ channel, K^+^ (V channel), SK_Ca_ channel, Na channel (site 2), DA transporter, NE transporter, 5-HT transporter.

### Pharmacokinetics and bioanalysis

The pharmacokinetic of GSK1059865 was determined following intra-peritoneal administration to male CD rats (250–350 g, Charles River, Italy) as previously described [43]. GSK1059865 was dissolved in a vehicle composed of 5% dimethyl-sulphoxide/1.5% HPMC/0.15% SLS (Sodium Lauryl Sulphate) and containing 10% (w/v) D-Mannitol. This vehicle was also used for JNJ10397049 throughout the study. The compound was administered intraperitoneally at 10 or 30 mg/kg (vehicle volume 4 mL/kg, N = 3 per dose). Blood samples were collected via femoral vein at intervals up to 5 h after administration. Brain samples were collected at the end of the experiment. The concentration of GSK1059865 was determined using a method based on protein precipitation followed by HPLC-MS/MS analysis. Non-compartmental pharmacokinetic parameters were obtained from the blood concentration-time profiles using the software package WinNonlin v.4.0 (Pharsight Corp. Mountain View, CA, USA). *In vitro* blood and brain unbound fractions were determined incubating GSK1059865 at 1 µg/mL in a 96-well equilibrium dialysis apparatus (HTDialysis, Gales Ferry, CT) using the method described by Kalvass and colleagues [44]. Theoretical receptor occupancy for GSK1059865 was calculated applying the law of mass action as described in greater detail elsewhere [43], [45]: 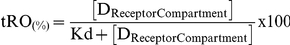
assuming unbound blood and brain concentration corresponding to average exposure (C_average._). Pharmacokinetic parameters are expressed as mean ± standard error of the mean.

### Pharmacological Magnetic Resonance Imaging

The studies were performed on Male Sprague-Dawley rats (Charles River, weight range 250–344 grams). Animals had free access to standard rat chow and tap water and were housed in groups of 5. Room temperature (20–22°C), relative humidity (45–65%) and dark-light cycles (12 h each, lights on at 06:00 h) were automatically controlled.

Animal preparation/monitoring and MRI acquisition parameters have been extensively described in previous papers [25], [46], [47]. Briefly, rats were anaesthetized with 3% halothane, tracheotomised and artificially ventilated with a mechanical respirator under neuromuscular blockade (D-tubocurarine 0.25 mg/kg/h). The left femoral artery and vein were cannulated for compound administration and continuous measurement of arterial blood pressure (MABP). A cannula was also inserted intraperitoneally for drug pre-treatment. After surgery the rat was secured on a custom-made holder and halothane level set to 0.8%. The ventilation parameters were adjusted to maintain physiological arterial blood gases (p_a_CO_2_ and p_a_O_2_) levels according to measurements performed prior to and at the end of the fMRI time series acquisition (Table S1). No statistically significant difference between pre- and post-acquisition p_a_CO_2_ values for each of the amphetamine-challenged groups was found (one way ANOVA, p>0.68). Moreover, linear regression analysis did not show significant correlation between the amplitude of the relative cerebral blood volume (rCBV) response to amphetamine (expressed as 4–20 min post-injection average in the somatosensory cortex) and p_a_CO_2_ levels, when these were expressed as individual measurements (P>0.17, r<0.54), or pre- and post- acquisition difference (P>.67, r<.18). P_a_O_2_ levels were higher than 90 mmHg in all subjects, corresponding to > 98% hemoglobin saturation.

The body temperature of all subjects was maintained within physiological range (37±0.8°C) throughout the experiment, by using a water heating system incorporated in the stereotactic holder. MABP was monitored continually through the femoral artery at a sampling rate of 50 Hz and re-binned on 42 s bins to match temporal resolution of phMRI timeseries. At the end of the experiment, the animals were euthanized with an overdose of anaesthetic followed by cervical dislocation.

### rCBV measurement

MRI data were acquired using a Bruker Avance 4.7 Tesla system, a 72 mm birdcage resonators for radiofrequency pulse transmit and a curved quadrature receive coil. The MR acquisition for each subject comprised T_2_-weighted anatomical images using the RARE sequence (TR_eff_  = 5000 ms, TE_eff_  = 76 ms, RARE factor 8, FOV 40 mm, 256×256 matrix, 16 contiguous 1 mm slices) followed by a time series acquisition with the same spatial coverage and similar parameters (TR_eff_  = 2700 ms, TE_eff_  = 110 ms, RARE factor 32), but with a lower in-plane spatial resolution (128×128) giving a functional pixel volume of ∼0.1 mm^3^. Two successive scans were averaged for a resulting time resolution of 42 s. Total MRI time-series acquisition time was 77-min (110 repetitions) for all subjects. Following five reference images, 2.67 ml/kg of the blood pool contrast agent Endorem (Guerbet, France) was injected so that subsequent signal changes would reflect alterations in relative cerebral blood volume (rCBV) [28]. After an equilibration period of 15 min (22 images) each subject received an intraperitoneal pre-treatment followed by an acute intravenous challenge with d-amphetamine (or vehicle) 30 min later (image 62).

GSK1059865 and JNJ10397049 were used in two independent experiments.

#### OX1R antagonist study (GSK1059865)

Rats were randomly assigned to one of the two following groups:

- intraperitoneal (IP) pretreatment with vehicle (4 ml/kg) followed by intravenous challenge with d-amphetamine sulphate (SIGMA, 1 mg/kg, 1 ml/rat IV) 30 min later (group #1; N = 10)
- IP pretreatment with GSK1059865A (30 mg/kg) followed by intravenous challenge with d-amphetamine sulphate (1 mg/kg) 30 min later (group #2, N = 8)

#### OX2R antagonist study (JNJ10397049)

Rats were randomly assigned to one of the two following groups:

- IP pretreatment with vehicle (4 ml/kg) and intravenous challenge with d-amphetamine sulphate (1 mg/kg, 1 ml/rat IV) 30 min later (group #3, N = 9)
- IP pretreatment with JNJ10397049 (50 mg/kg) and intravenous challenge with d-amphetamine sulphate (1 mg/kg) 30 min later (group #4, N = 8)

An additional group of subjects pre-treated with vehicle and challenged with saline (1 ml/kg) was acquired to serve as reference rCBV baseline for both studies (group #5, N = 6).

All compounds were injected at a rate of 1 ml/min. Compound injection was followed by administration of 0.4 ml of saline to flush the intravenous line.

The dose chosen for d-amphetamine sulphate was based on previously published *in vivo* studies [18]. This dose elicits robust and reproducible brain activation without producing blood pressure alterations exceeding the cerebral blood flow autoregulatory range under halothane anaesthesia [19], [48], and is comparable to the dose that was shown to increase Fos expression in orexin neurons [16]. The dose used for this study does not induce “ceiling” rCBV responses, as previous studies using an identical protocol showed region-specific potentiation of D-amphetamine responses upon prior pharmacological modulation of dopamine receptors [18], [20].

### phMRI data analysis

rCBV time series image data for each experiment were analyzed within the framework of the general linear model (GLM) to obtain Z statistic maps [49]. The maps thus obtained were used to guide the selection of activated regions for subsequent volume of interest (VOI)–based quantification and comparison of efficacy of treatments.

Signal intensity changes in the time series were converted into fractional rCBV on a pixel-wise basis, using a constrained exponential model of the gradual elimination of contrast agent from the blood pool [50]. Individual subjects in each study were spatially normalized by a 9-degree–of-freedom affine transformation mapping their T_2_-weighted anatomical images to a stereotaxic rat brain MRI template set and applying the resulting transformation matrix to the accompanying rCBV time series.

rCBV time series for the amphetamine challenge (groups 1–4) were calculated covering 8.4 minute (12 timepoints) pre-challenge baseline and 21.0 minute (30 timepoints) post-challenge windows, normalized to a common injection time point. Image based time series analysis was carried out using FEAT (FMRI Expert Analysis Tool) Version 5.63, part of FSL (FMRIB's Software Library, www.fmrib.ox.ac.uk/fsl) with 0.8 mm spatial smoothing (≈2.5× in-plane voxel dimension) and using a model function identified by Wavelet Cluster Analysis (WCA) across all animals in the cohort, capturing the temporal profile of the signal change induced by the amphetamine challenge in each group [51]. The design matrix also included the temporal derivative of this regressor and a linear ramp (both orthogonalised to the regressor of interest) with the aim to capture additional variance due to slight deviations in individual subjects or brain regions from the signal model time course as described more in detail elsewhere [52]. The model functions obtained for the two studies were very similar and captured very well the sustained positive rCBV changes produced by the drug challenge (Figure S1). The coefficients of the model function thus provided a map of rCBV response amplitude for each injection in each subject. Higher-level group comparisons were carried out using FLAME (FMRIB's Local Analysis of Mixed Effects); Z (Gaussianised T/F) statistic images were thresholded using clusters determined by Z>2.33 (study 1) or Z>1.96 (study 2) and a corrected cluster significance threshold of p = 0.01 [49], [53]. To rule out the presence of significant short-lived contributions to the pattern of activation produced by amphetamine in the different groups, we performed an additional GLM analysis using a regressor that we identified retaining only high temporal frequency components in the WCA analysis with the aim to capture subtle short-lived responses similar to the transient rCBV depression produced by intravenous vehicle. This analysis did not highlight any significantly activated or deactivated voxel vs. vehicle-vehicle baseline for any of the groups analyzed (Z>1.6, cluster correction p = 0.05).

VOI time courses for the amphetamine challenge were extracted from unsmoothed rCBV time series data using a 3D digital reconstruction of a rat brain atlas [54] co-registered with the MRI template, using software written in IDL (Research Systems Inc., Boulder, Colorado). A list of the VOIs examined and their anatomical definitions can be found in [25]. For each VOI time course, the average rCBV over a 6 min time window covering the peak of the response to amphetamine (4–10 min post-injection) was used as a summary statistic of the relative change. The effect of OX1R antagonist pretreatment on the magnitude of average rCBV in different VOIs was assessed by a one-way ANOVA followed by Fishers's LSD test versus vehicle-amphetamine (groups 1 and 3, respectively). Results are quoted and displayed as mean ± SEM.

As subjects received the pre-treatment during the phMRI time-series acquisition, VOI time-courses of pre-treatment *per se* were examined to exclude the presence of rCBV “ceiling” or “floor” effects that might have influenced or prevented the subsequent response to amphetamine. To this end, rCBV time-courses were also calculated for the pre-treatment over a time-window covering 5.6 minutes (8 timepoints) pre-injection baseline and 25.2 minutes (36 timepoints) post-injection window normalized to a common injection time point. VOI timecourses were extracted from unsmoothed rCBV time series in the same regions examined for the amphetamine challenge.

### Sleep recording and analysis

Male Sprague Dawley rats (275–300 g, Charles River Italy) were housed singly on 12 h light dark cycle (15:00–03:00 light) one week prior to surgery. Access to food and water was allowed *ad libitum*. In order to collect the biopotential signals, a miniature multichannel telemetric transmitter (TL10M3-F40-EET, Data Sciences Int.) was implanted intraperitoneally into the animals. To allow recording of cortical electroencephalogram (EEG), two electrodes were fixed permanently to the skull with dental cement. The electrodes were placed in direct contact with the *dura mater* through two drilled holes on the fronto-parietal region. Two additional electrodes were fixed to the skeletal muscles of the neck, to allow electromyogram (EMG) recordings. Although implanted animals demonstrated a normal behavioural repertoire immediately after surgery, a three weeks time period was allowed prior to experimental utilisation in order to allow normal sleep patterns to be re-established.

For the duration of the test period freely moving animals remained in their home cages on individual receivers. EEG and EMG signals were recorded continuously using DSI Dataquest® A.R.T. The EEG trace, divided into ten second epochs, was digitally transformed (FFT transformation) to provide the power spectra of δ, θ, α and β bands so to distinguish three different activity patterns in the rat (awake, NREM sleep and REM sleep). The markers assigned by the automated scoring system (*Sleep stage®*, DSI) were transferred to the EEG digital signal and subsequently confirmed by visual examination of the EEG and EMG traces by trained operators, blind to the drug treatment.

Analysis of sleep parameters included: time spent wake, NREM sleep, REM sleep and total sleep time (TST).

Drug studies were carried out according to a randomised paired crossover design where each animal received control and drug treatments in separate experimental sessions. Animals were treated with GSK1059865 or JNJ10397049 or vehicle six hours into the dark phase (Circadian time (CT) 18). At this point in the light/dark cycle the concentration of the orexin-A peptide is high, thus maximising the pharmacological window of effective antagonism [21]. Recordings were made over a 5-hour test period. Results were expressed as mean value ± S.E.M. Statistical analysis was performed by a one-way analysis of variance followed by Dunnett's test (summary measurements) or Newmann-Keuls (individual time-points in NREM and REM timecourses).

### Conditioned place –preference (CPP)

The studies were performed on Male Sprague-Dawley rats (155–165 g upon arrival, Charles River, Como, Italy). Animals were housed two per cage and kept on a 12 hours lights on/12 hours lights off schedule and food and water were available *ad libitum*. An automated, three-chambered CPP apparatus was used as previously described [55]. The two pairing chambers of the apparatus were identical in dimensions and separated by removable plexiglass guillotine doors. The pairing chambers were fitted with different visual and tactile cues. The cues were balanced to avoid the development of side preferences prior to conditioning. Each of the pairing chambers had an infrared micro-beam that was wired to an automatic timer to measure the time spent in each chamber.

CPP acquisition and expression were both established and assessed as previously

described [55]. Briefly, animals were habituated to the experimental room and handled for 5 days prior to the CPP experiments. Prior to the start of the CPP experiments, the animals were allowed to freely explore the apparatus for 15 min. Animals were then randomly divided into 6 groups (n = 10 each). One group received 4 pairings (one injection per day) with vehicle (water, vehicle-vehicle), while the remaining 5 groups received 4 pairings with vehicle followed by 15 mg/kg i.p. of cocaine (vehicle-cocaine). The animals were confined to one of two conditioning chambers for 30 min during each pairing session. On the test day the animals received either vehicle or GSK1059865A (3, 10 or 30 mg/kg i.p) 30 minutes prior to the behavioural test. The overall pairing scheme used is described below:

*Pairings treatments on the test day*

Vehicle-Vehicle Vehicle i.p.

Vehicle-Cocaine Vehicle i.p.

Vehicle-Cocaine 3 mg/kg of GSK1059865 i.p.

Vehicle- Cocaine 10 mg/kg of GSK1059865 i.p.

Vehicle- Cocaine 30 mg/kg of GSK1059865 i.p.

The animals were allowed access to the entire apparatus for 15 min, and the data were expressed as the raw time spent in each chamber. Statistical analysis was performed with GB-STAT, version 6.5 (Dynamics Microsystsms, Inc., Silver Spring, MD, USA, 1997) using a one-way analysis of variance (ANOVA) followed by a Student- Newmans-Keuls test.

## Supporting Information

Figure S1 Time–profile of the temporal components identified with WCA [52] in groups 1–2 (Regr 1), and 3–4 (Regr 2). The regressors were used as model function for GLM analysis as detailed in the methods section.
(TIF)

Figure S2 Temporal profile of mean arterial blood pressure (MABP) produced by intraperitoneal pretreatment (Left) or amphetamine challenge (Right); Data are plotted as mean±SEM within each group. Upper and lower cerebral blood flow autoregulation range under halothane anaesthesia are illustrated by dashed lines at 120 and 60 mmHg, respectively [19]; Ox1R_ant_: GSK1059865 30 mg/kg i.p.; Ox2R_ant:_: JNJ10397049 50 mg/kg i.p.
(TIF)

Figure S3 Effect of pretreatment on basal CBV in representative brain regions. Data are plotted as mean±SEM within each group. Ox1R_ant_: GSK1059865 30 mg/kg i.p.; [Mt Ctx: primary motor cortex; SS Ctx: somatosensory cortex; mPFC: medial prefrontal cortex; Ins Ctx; insular cortex; Thal; thalamus; Hipp: hippocampus].
(TIF)

Figure S4 Effect of pretreatment on basal rCBV in representative brain regions. Data are plotted as mean±SEM within each group. Ox2R_ant_: JNJ10397049 50 mg/kg i.p. [Mt Ctx: primary motor cortex; SS Ctx: somatosensory cortex; mPFC: medial prefrontal cortex; Ins Ctx; insular cortex; Thal; thalamus; Hipp: hippocampus].
(TIF)

Table S1 Abbreviations: PaCO2 - partial pressure of arterial CO2; Pre and Post: measurements performed prior to and after the fMRI timeseries, respectively. Values presented as mean ± SEM. Ox1ant-: GSK1059865 30 mg/kg i.p.; Ox2ant:: JNJ10397049 50 mg/kg i.p.
(DOC)

## References

[1] Sakurai T (1998). Orexins and orexin receptors: a family of hypothalamic neuropeptides and G protein-coupled receptors that regulate feeding behavior.. Cell.

[2] Sakurai T (2007). The neural circuit of orexin (hypocretin): maintaining sleep and wakefulness.. Nat Rev Neurosci.

[3] Funato H, Tsai AL, Willie JT, Kisanuki Y, Williams SC (2009). Enhanced Orexin Receptor-2 Signaling Prevents Diet-Induced Obesity and Improves Leptin Sensitivity.. Cell Metabolism.

[4] Willie JT, Chemelli RM, Sinton CM, Tokita S, Williams SC (2003). Distinct narcolepsy syndromes in Orexin receptor-2 and Orexin null mice: molecular genetic dissection of Non-REM and REM sleep regulatory processes.. Neuron.

[5] Lee MG, Hassani OK, Jones BE (2005). Discharge of Identified Orexin/Hypocretin Neurons across the Sleep-Waking Cycle.. Journal of Neuroscience.

[6] Aston-Jones G, Smith RJ, Moorman DE, Richardson KA (2009). Role of lateral hypothalamic orexin neurons in reward processing and addiction.. Neuropharmacology.

[7] Marcus JN (2001). Differential expression of orexin receptors 1 and 2 in the rat brain.. J Comp Neurol.

[8] Chemelli RM (1999). Narcolepsy in orexin knockout mice: molecular genetics of sleep regulation.. Cell.

[9] Dugovic C, Shelton JE, Aluisio LE, Fraser IC, Jiang X (2009). Blockade of Orexin-1 Receptors Attenuates Orexin-2 Receptor Antagonism-Induced Sleep Promotion in the Rat.. J Pharmacol Exp Ther.

[10] Harris GC, Wimmer M, ston-Jones G (2005). A role for lateral hypothalamic orexin neurons in reward seeking.. Nature.

[11] Hollander JA, Lu Q, Cameron MD, Kamenecka TM, Kenny PJ (2008). Insular hypocretin transmission regulates nicotine reward.. PNAS.

[12] Brisbare-Roch C (2007). Promotion of sleep by targeting the orexin system in rats, dogs and humans.. Nature Med.

[13] Coleman PJ, Renger JJ (2010). Orexin receptor antagonists: a review of promising compounds patented since 2006.. Expert Opin Ther Pat.

[14] McAtee LC, Sutton SW, Rudolph DA, Li X, Aluisio LE (2004). Novel substituted 4-phenyl-[1,3]dioxanes: potent and selective orexin receptor 2 (OX2R) antagonists.. Bioorganic & Medicinal Chemistry Letters.

[15] Alvaro G, Amantini D, Stasi L (2009). Pyridine derivatives used to treat Orexin related disorders..

[16] Fadel J, Bubser M, Deutch AY (2002). Differential activation of orexin neurons by antipsychotic drugs associated with weight gain.. J Neurosci.

[17] Estabrooke IV, McCarthy MT, Ko E, Chou TC, Chemelli RM (2001). Fos expression in orexin neurons varies with behavioral state.. J Neurosci.

[18] Schwarz A, Gozzi A, Reese T, Bertani S, Crestan V (2004). Selective dopamine D(3) receptor antagonist SB-277011-A potentiates phMRI response to acute amphetamine challenge in the rat brain.. Synapse.

[19] Gozzi A, Ceolin L, Schwarz A, Reese T, Bertani S (2007). A multimodality investigation of cerebral haemodynamics and autoregulation in phMRI.. Magnetic Resonance Imaging,.

[20] Micheli F, Bonanomi G, Blaney FE, Braggio S, Capelli AM (2007). 1,2,4-triazol-3-yl-thiopropyl-tetrahydrobenzazepines: a series of potent and selective dopamine D(3) receptor antagonists.. J Med Chem.

[21] Yoshida Y (2001). Fluctuation of extracellular hypocretin-1 (orexin A) levels in the rat in relation to the light-dark cycle and sleep-wake activities.. Eur J Neurosci.

[22] Bardo MT, Bevins RA (2000). Conditioned place preference: what does it add to our preclinical understanding of drug reward?. Psychopharmacology.

[23] Everitt BJ, Morris KA, O'Brien A, Robbins TW (1991). The basolateral amygdala-ventral striatal system and conditioned place preference: Further evidence of limbic-striatal interactions underlying reward-related processes.. Neuroscience.

[24] Schwarz AJ, Gozzi A, Reese T, Bifone A (2007). In vivo mapping of functional connectivity in neurotransmitter systems using pharmacological MRI.. NeuroImage.

[25] Gozzi A, Large C, Schwarz A, Bertani S, Crestan V (2008). Differential Effects of Antipsychotic and Glutamatergic Agents on the phMRI Response to Phencyclidine.. Neuropsychopharmacology.

[26] Sheth SA, Nemoto M, Guiou M, Walker M, Pouratian N (2004). Columnar Specificity of Microvascular Oxygenation and Volume Responses: Implications for Functional Brain Mapping.. Journal of Neuroscience.

[27] Chen Y-CI, Mandeville JB, Nguyen TV, Talele A, Cavagna F (2001). Improved Mapping of Pharmacologically Induced Neuronal Activation Using the IRON Technique with Superparamagnetic Blood Pool Agents.. Journal of Magnetic Resonance Imaging.

[28] Mandeville JB, Marota JJA, Kosofsky BE, Keltner JR, Weissleder R (1998). Dynamic functional imaging of relative cerebral blood volume during rat forepaw stimulation.. Magn Reson Med.

[29] Maquet P (2000). Functional neuroimaging of normal human sleep by positron emission tomography.. J Sleep Res.

[30] Smith MI, Piper DC, Duxon MS, Upton N (2003). Evidence implicating a role for orexin-1 receptor modulation of paradoxical sleep in the rat.. Neuroscience Letters.

[31] Willie JT, Chemelli RM, Sinton CM, Yanagisawa M (2001). To eat or to sleep? Orexin in the regulation of feeding and wakefulness.. Annu Rev Neurosci.

[32] Knutson B, Gibbs S (2007). Linking nucleus accumbens dopamine and blood oxygenation.. Psychopharmacology.

[33] Fadel J, Deutch AY (2002). Anatomical substrates of orexin-dopamine interactions: lateral hypothalamic projections to the ventral tegmental area.. Neuroscience.

[34] Peyron C (1998). Neurons containing hypocretin (orexin) project to multiple neuronal systems.. J Neurosci.

[35] Lee MG, Hassani OK, Jones BE (2005). Discharge of identified orexin/hypocretin neurons across the sleep-waking cycle.. J Neurosci.

[36] Mileykovskiy BY, Kiyashchenko LI, Siegel JM (2005). Behavioral Correlates of Activity in Identified Hypocretin/Orexin Neurons.. Neuron.

[37] Scammell TE, Saper CB (2005). Orexin, drugs and motivated behaviors.. Nat Neurosci.

[38] Naqvi NH, Bechara A (2009). The hidden island of addiction: the insula.. Trends in Neurosciences.

[39] Gompf H, Chen J, Sun Y, Yanagisawa M, Aston-Jones G (2009). Halothane-induced Hypnosis Is Not Accompanied by Inactivation of Orexinergic Output in Rodents.. Anesthesiology.

[40] Brandish PE, Hill LA, Zheng W, Scolnick EM (2003). Scintillation proximity assay of inositol phosphates in cell extracts: High-throughput measurement of G-protein-coupled receptor activation.. Analytical Biochemistry.

[41] Kenakin T, Jenkinson S, Watson C (2006). Determining the Potency and Molecular Mechanism of Action of Insurmountable Antagonists.. J Pharmacol Exp Ther.

[42] Runlakshana O, Schild H (1959). Some quantitative uses of drug antagonists.. Br J Pharmacol Chemother.

[43] Ferrari L, Crestan V, Sabattini G, Vinco F, Fontana S (2010). Brain penetration of local anaesthetics in the rat: Implications for experimental neuroscience.. Journal of Neuroscience Methods.

[44] Kalvass JC, Maurer TS (2002). Influence of nonspecific brain and plasma binding on CNS exposure: implications for rational drug discovery.. Biopharm Drug Dispos.

[45] Read KD, Braggio S (2010). Assessing brain free fraction in early drug discovery.. Expert Opinion on Drug Metabolism & Toxicology.

[46] Gozzi A, Herdon H, Schwarz A, Bertani S, Crestan V (2008). Pharmacological stimulation of NMDA receptors via co-agonist site suppresses fMRI response to phencyclidine in the rat.. Psychopharmacology.

[47] Gozzi A, Crestan V, Turrini G, Clemens M, Bifone A (2010). Antagonism at serotonin 5-HT2A receptors modulates functional activity of frontohippocampal circuit.. Psychopharmacology.

[48] Zaharchuk G, Mandeville JB, Bogdanov AA, Weissleder R, Rosen BR (1999). Cerebrovascular dynamics of autoregulation and hypoperfusion. An MRI study of CBF and changes in total and microvascular cerebral blood volume during hemorrhagic hypotension.. Stroke.

[49] Worsley KJ, Evans AC, Marrett S, Neelin P (1992). A three-dimensional statistical analysis for CBF activation studies in human brain.. J Cereb Blood Flow Metab.

[50] Schwarz AJ, Reese T, Gozzi A, Bifone A (2003). Functional MRI using intravascular contrast agents: detrending of the relative cerebrovascular (rCBV) time course.. Magn Reson Imaging.

[51] Schwarz AJ, Danckaert A, Reese T, Gozzi A, Paxinos G (2006). A stereotaxic MRI template set for the rat brain with tissue class distribution maps and co-registered anatomical atlas: application to pharmacological MRI.. NeuroImage.

[52] Schwarz AJ, Whitcher B, Gozzi A, Reese T, Bifone A (2006). Study-level wavelet cluster analysis and data-driven signal models in pharmacological MRI.. J Neurosci Methods.

[53] Friston KJ, Jezzard P, Turner R (1994). Analysis of functional MRI time-series.. Human Brain Mapping.

[54] Paxinos G, Watson C (1998). The Rat Brain in Stereotactic Coordinates..

[55] Horan B, Gardner EL, Ashby CR (2000). Enhancement of conditioned place preference response to cocaine in rats following subchronic administration of 3, 4-methylenedioxymethamphetamine (MDMA).. Synapse.

