# Supplementary figures and images for: Functional Magnetic Resonance Imaging Reveals Different Neural Substrates for the Effects of Orexin-1 and Orexin-2 Receptor Antagonists
Source: PLoS One. 2011 Jan 28;6(1):e16406. doi: 10.1371/journal.pone.0016406 (PMC3030585; doi:10.1371/journal.pone.0016406)

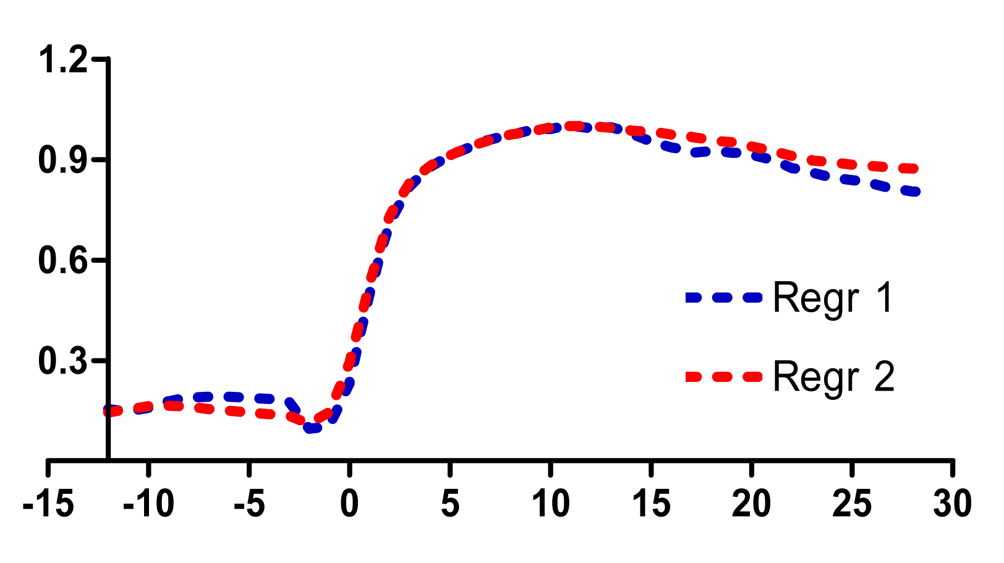

Supplement: Figure S1 — Time–profile of the temporal components identified with WCA [52] in groups 1–2 (Regr 1), and 3–4 (Regr 2). The regressors were used as model function for GLM analysis as detailed in the methods section. (TIF) [file pone.0016406.s001.tif]

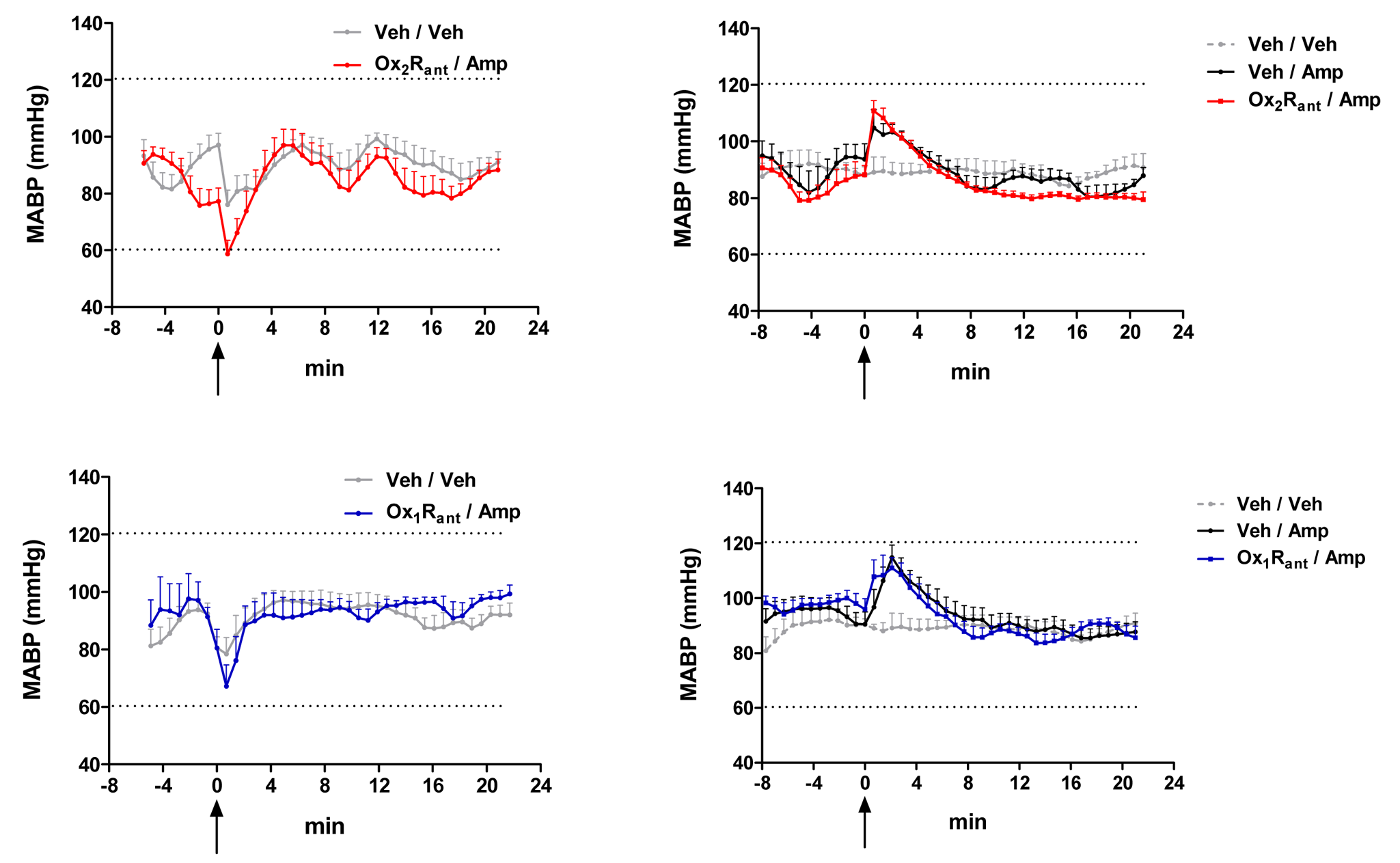

Supplement: Figure S2 — Temporal profile of mean arterial blood pressure (MABP) produced by intraperitoneal pretreatment (Left) or amphetamine challenge (Right); Data are plotted as mean±SEM within each group. Upper and lower cerebral blood flow autoregulation range under halothane anaesthesia are illustrated by dashed lines at 120 and 60 mmHg, respectively [19]; Ox1Rant: GSK1059865 30 mg/kg i.p.; Ox2Rant:: JNJ10397049 50 mg/kg i.p. (TIF) [file pone.0016406.s002.tif]

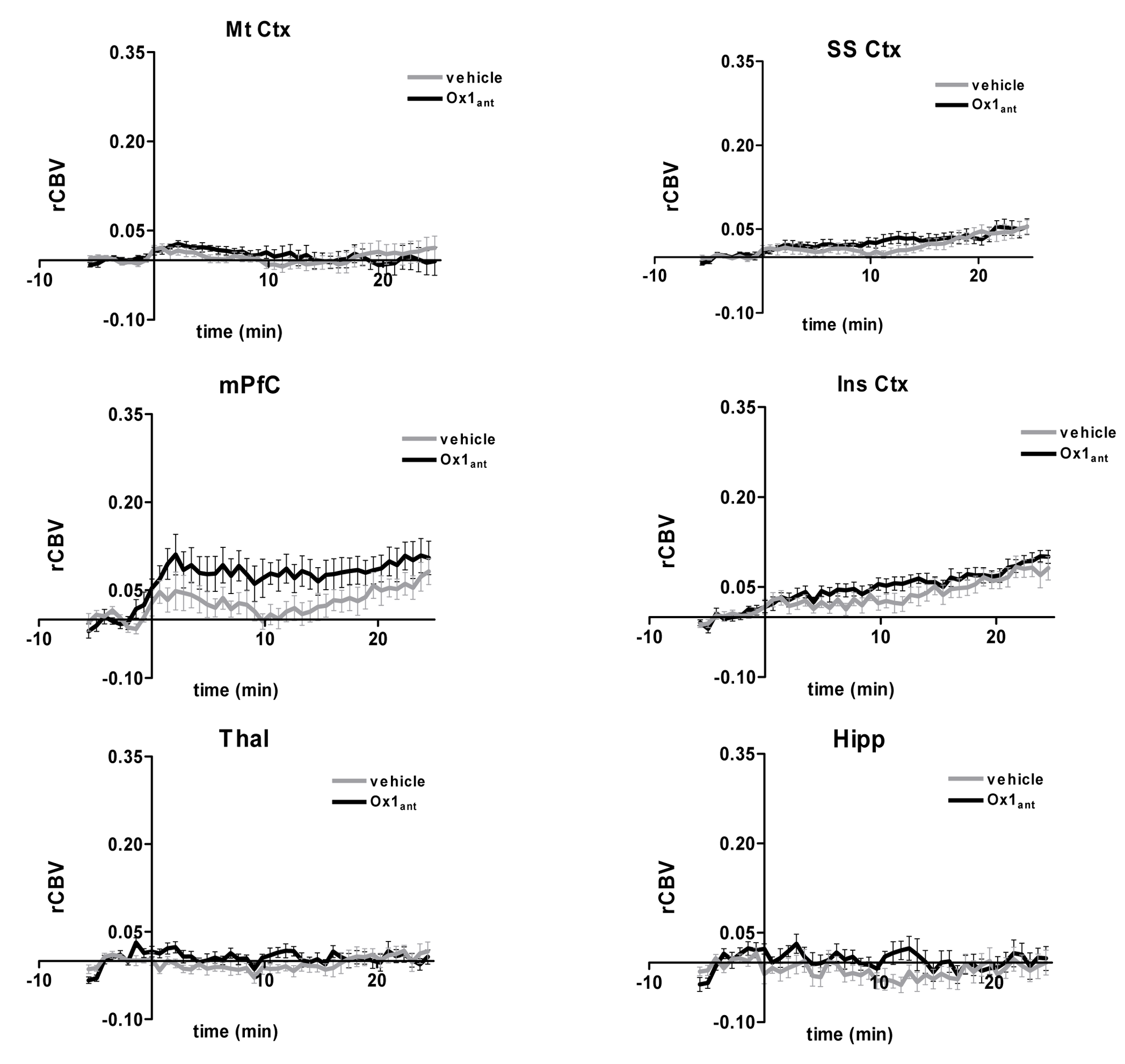

Supplement: Figure S3 — Effect of pretreatment on basal CBV in representative brain regions. Data are plotted as mean±SEM within each group. Ox1Rant: GSK1059865 30 mg/kg i.p.; [Mt Ctx: primary motor cortex; SS Ctx: somatosensory cortex; mPFC: medial prefrontal cortex; Ins Ctx; insular cortex; Thal; thalamus; Hipp: hippocampus]. (TIF) [file pone.0016406.s003.tif]

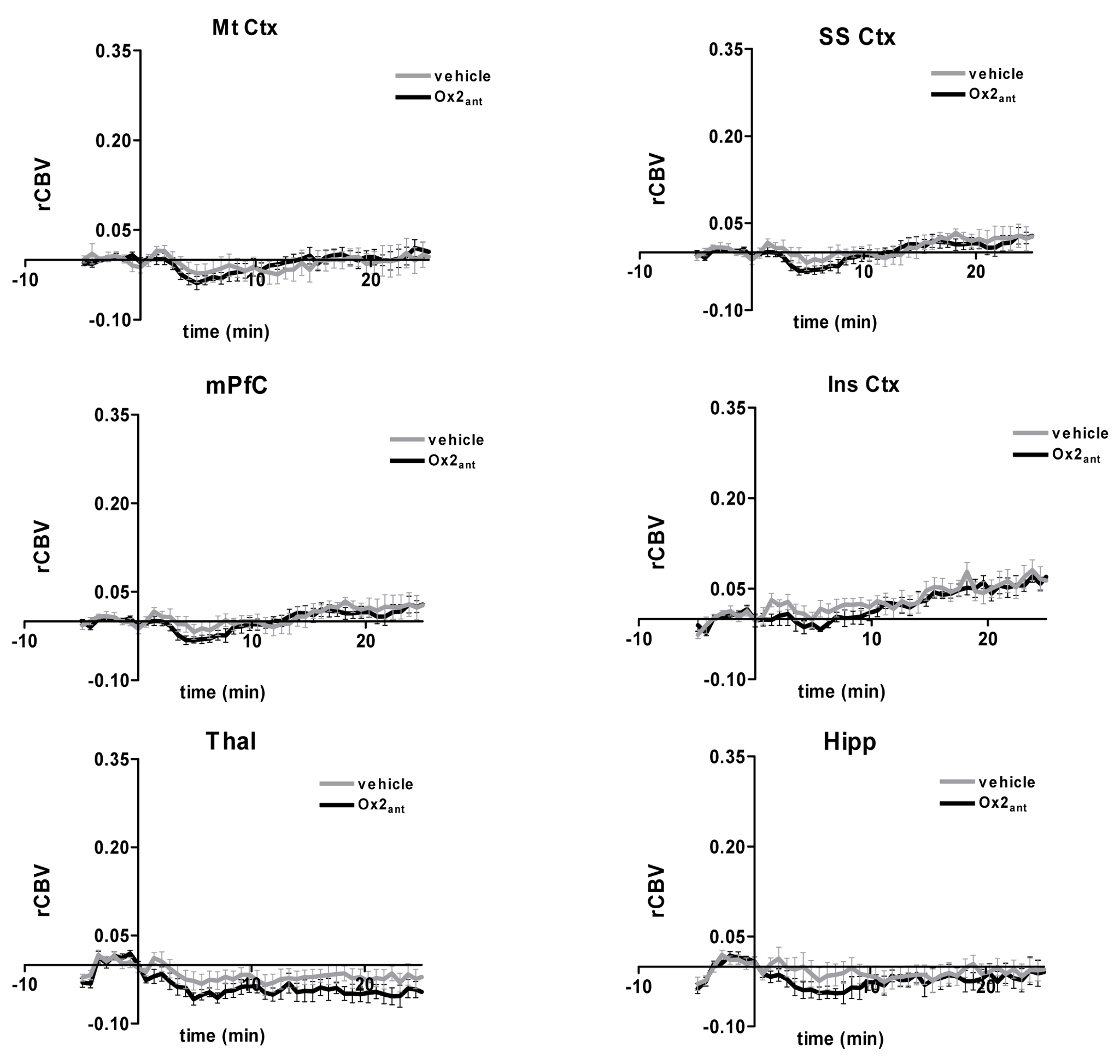

Supplement: Figure S4 — Effect of pretreatment on basal rCBV in representative brain regions. Data are plotted as mean±SEM within each group. Ox2Rant: JNJ10397049 50 mg/kg i.p. [Mt Ctx: primary motor cortex; SS Ctx: somatosensory cortex; mPFC: medial prefrontal cortex; Ins Ctx; insular cortex; Thal; thalamus; Hipp: hippocampus]. (TIF) [file pone.0016406.s004.tif]
