# Supplementary material for: Functional Magnetic Resonance Imaging Reveals Different Neural Substrates for the Effects of Orexin-1 and Orexin-2 Receptor Antagonists
Source: PLoS One. 2011 Jan 28;6(1):e16406. doi: 10.1371/journal.pone.0016406 (PMC3030585; doi:10.1371/journal.pone.0016406)
Supplement: Table S1 — Abbreviations: PaCO2 - partial pressure of arterial CO2; Pre and Post: measurements performed prior to and after the fMRI timeseries, respectively. Values presented as mean ± SEM. Ox1ant-: GSK1059865 30 mg/kg i.p.; Ox2ant:: JNJ10397049 50 mg/kg i.p. (DOC) [file pone.0016406.s005.doc]

**Table S1**

*Arterial CO_2_ measurements*

| Group | p_a_CO_2_ (mmHg) – Pre | p_a_CO_2_ (mmHg) - Post |
| --- | --- | --- |
| #1 Veh – Veh (n=6) | 39.7±1.9 | 36.5±1.9 |
| #2 Veh – Amp (n=10) | 34.1±2.3 | 39.1±2.5 |
| #3 Ox1_ant_ – Amp(n=8) | 33.0±1.3 | 39.4±2.4 |
| #4 Veh – Amp 4 (n=9) | 32.7±1.5 | 38.7±1.7 |
| #5 Ox2_ant_ – Amp (n=8) | 33.0±1.5 | 39.6±2.1 |

Abbreviations: P_a_CO_2_ - partial pressure of arterial CO_2_; Pre and Post: measurements performed prior to and after the fMRI timeseries, respectively. Values presented as mean ± SEM. Ox1_ant_-: GSK1059865 30 mg/kg i.p.; Ox2_ant_:: JNJ10397049 50 mg/kg i.p.
